# Placental H3K27me3 establishes female resilience to prenatal insults

**DOI:** 10.1038/s41467-018-04992-1

**Published:** 2018-07-02

**Authors:** Bridget M. Nugent, Carly M. O’Donnell, C. Neill Epperson, Tracy L. Bale

**Affiliations:** 1 Department of Pharmacology, University of Maryland School of Medicine Health Sciences Facility, III 670 W. Baltimore Street, Baltimore, MD 21201 USA; 2 Department of Biomedical Sciences, University of Pennsylvania 380 S. University Ave, Philadelphia, PA 19104 USA; 3 Department of Psychiatry, University of Pennsylvania, 3535 Market Street, 3rd Floor, Philadelphia, PA 19104 USA

## Abstract

Although sex biases in disease presentation are well documented, the mechanisms mediating vulnerability or resilience to diseases are unknown. In utero insults are more likely to produce detrimental health outcomes for males versus females. In our mouse model of prenatal stress, male offspring experience long-term dysregulation of body weight and hypothalamic pituitary adrenal stress axis dysfunction, endophenotypes of male-biased neurodevelopmental disorders. Placental function is critical for healthy fetal development, and we previously showed that sex differences in placental O-linked *N*-acetylglucosamine transferase (OGT) mediate the effects of prenatal stress on neurodevelopmental programming. Here we show that one mechanism whereby sex differences in OGT confer variation in vulnerability to prenatal insults is by establishing sex-specific trophoblast gene expression patterns and via regulation of the canonically repressive epigenetic modification, H3K27me3. We demonstrate that high levels of H3K27me3 in the female placenta create resilience to the altered hypothalamic programming associated with prenatal stress exposure.

## Abstract (web summary)

Sex differences in placental O-linked *N*-acetylglucosamine transferase (OGT) activity mediate the effects of prenatal stress on neurodevelopmental programming. Here authors provide evidence that OGT confers variation in vulnerability to prenatal insults by establishing sex-specific trophoblast gene expression via regulation of H3K27me3.

## Introduction

Identifying the developmental factors that promote disease susceptibility and resilience is critical to elucidating the etiology of disease prevalence, severity, and symptomology. Medical research has only recently begun to appreciate that biological sex is one of the greatest predictors of disease risk and treatment outcome. Sex biases in prevalence and symptomology are pervasive among some of the most prevalent health conditions affecting Western societies today—from hypertension to diabetes, arthritis, and cancers^1^. Neurological and psychiatric disorders are especially noteworthy in their sex biases with post-pubertal onset diseases, such as depression and anxiety, preferentially affecting females, and neurodevelopmental disorders, including autism spectrum disorders, early onset schizophrenia, and attention deficit hyperactivity disorders presenting at significantly higher rates in boys^2–4^. At birth, males are more likely to be admitted to the neonatal intensive care unit and have higher infant mortality than females^5,6^. In addition, males are more sensitive to prenatal insults, such as gestational stress, maternal infection, and drug exposure, which impart metabolic, cognitive, and behavioral deficits in boys and an increased lifetime risk for disease presentation^7^. These statistics support the likelihood that the prenatal environment imparts sex-specific, long-term health consequences whereby males are at greater risk for detrimental outcomes following in utero perturbations.

During early embryonic development, the placenta provides the developing fetus with the nutrients and growth factors necessary for development, through direct placental production and via appropriation from maternal circulation^8^. The placenta is largely comprised of cells originating from the fetally derived trophoblast lineage, which are the first cells to differentiate after fertilization and form the outer blastocyst layer in the early embryo^9^. The trophoblast lineage’s fetal origin means that the placenta reflects fetal sex chromosome complement. Consequently, X- and Y-linked genes have the potential to introduce sex differences in placental development and function. Studies examining human placenta and utilizing animal models have demonstrated that placental function is sensitive to changes in the maternal milieu as evidenced by fluctuations in gene expression, placental morphology and weight in response to nutrient cues, drug and alcohol intake, infection and stress^10–21^. The energetically expensive developing brain is likely especially sensitive to environmentally derived perturbations in placental function^8^. Consequently, sex differences in placental activity and responses to insults may contribute to increased male risk and female resilience, including to certain neurodevelopmental disorders.

Consistent with epidemiological evidence for greater prenatal sensitivity in males, our mouse model of prenatal stress in which pregnant females are exposed to chronic stress during the first week of gestation, produces male offspring with a phenotype of neuroendocrine dysregulation, including hyperactive hypothalamic pituitary adrenal (HPA) stress axis sensitivity and metabolic dysfunction^22,23^. Using a large-scale genomic screen at three gestational time points (E12.5, E15.5, and E18.5), we previously identified placental O-linked *N*-acetylglucosamine transferase (*Ogt*) as a biomarker of prenatal stress exposure in mice^24^. *Ogt* codes for an X-linked enzyme (OGT) that utilizes cellular nutrients, such as glucose, glutamine, ATP, and acetyl-CoA to O-glycNAcylate thousands of proteins and alter cellular function^25^. *Ogt* mRNA and protein levels are nearly twice as high in female placental tissue than in male tissue in both rodents and humans^24^. This already low level of *Ogt* in the male placenta is then further significantly decreased by prenatal stress^24^. We previously demonstrated that these low levels of placental *Ogt* were causal in determining neurodevelopmental vulnerability, as conditional, trophoblast-specific reduction of *Ogt* recapitulated the prenatal stress phenotype^26^. Further, males with placental *Ogt* deletion show large-scale changes in gene expression and reduction in mitochondrial function in the paraventricular nucleus of the hypothalamus (PVN), a critical neuroendocrine brain region that determines life-long stress responsivity^26^. Whole-brain microRNA profiles are also disrupted by placental *Ogt* reduction^24^. Taken together these results suggest that normal function of placental OGT and the subsequent trans-placental signals are critical for the developing brain, and that sex differences in placental OGT may mediate the increased male vulnerability to neurodevelopmental disorders.

OGT’s multitude of cellular functions suggests that small variations in its enzymatic activity could have dramatic effects on trophoblast cell function. For instance, OGT is known for directly and indirectly broadly control transcription by enhancing RNA polymerase II activity and histone modifications^25^. Thus significant differences in *Ogt* levels have the potential to determine sex-specific programs of trophoblast gene expression that may mediate sex differences in placental function and trans-placental signals relaying information regarding prenatal insults to the developing fetus. Recently, OGT was shown to guide broad epigenomic patterning by mediating histone methylation, including the ubiquitous repressive histone modification, H3K27me3, via structural stabilization of the histone methyltransferase, enhancer of zeste homolog 2 (Ezh2), a member of the polycomb repressive complex 2 (PRC2)^27^. Therefore, OGT and Ezh2-mediated changes in placental H3K27me3 may underlie large-scale sex differences and responses to prenatal perturbations, such as stress, by providing tighter control of transcriptional repression in one sex over the other.

Here we test the hypothesis that female prenatal resilience is driven by sex differences in placental transcriptional control, in part by the X-linked gene, *Ogt*, and its regulation of the histone repressive mark, H3K27me3. We find that levels of placental *Ogt* determine sex differences in fetally derived placental trophoblast transcriptome profiles associated with key developmental processes and shape genome-wide patterning of the ubiquitous epigenetic transcriptional repressive mark, H3K27me3.

## Results

### *Ogt* regulates sex differences in trophoblast gene expression

**Fig. 1:**
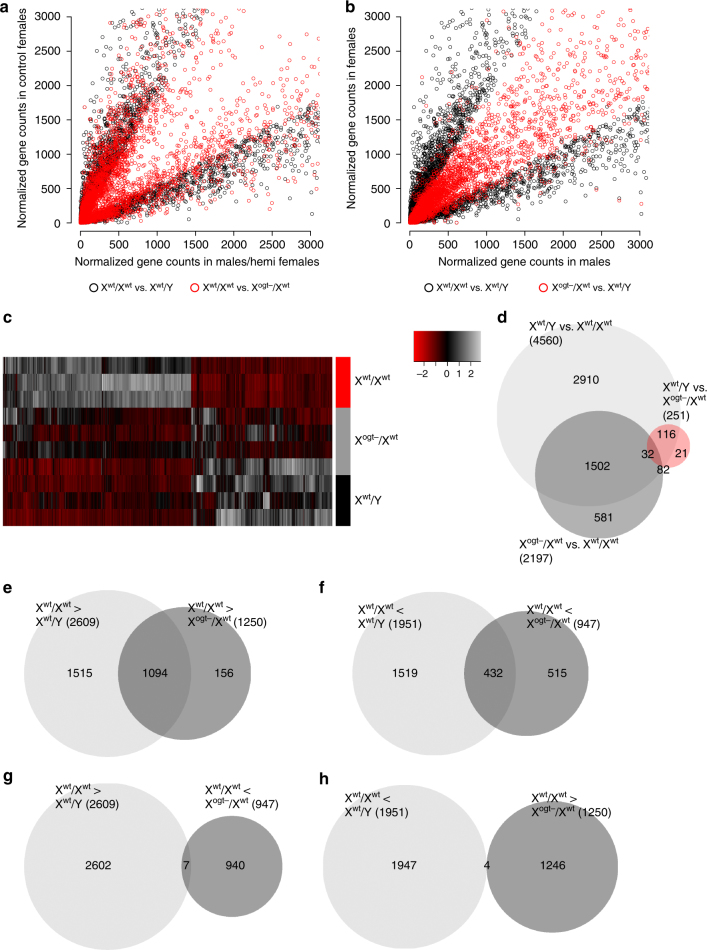
Sex differences in placental trophoblast and fetal hypothalamic gene expression are mediated by placental *Ogt* copy number. **a**, **b** Scatterplots of mRNAs with sex differences in trophoblast cell-specific expression at E12.5 (4560 genes, FDR adjusted *p* < 0.05). **a** Scatterplot comparing sex differences (black circles) with differences between X^wt^/X^wt^ and X^ogt−^/X^wt^ females (red circles). Trophoblast gene expression patterns with sex differences (black circles) are mimicked by *Ogt* reduction in female trophoblasts (red circles). **b** Scatterplot comparing trophoblast sex differences (black circles) with differences between X^ogt−^/X^wt^ and X^wt^/Y (red circles). Female patterns of gene expression are masculinized by *Ogt* reduction in female trophoblasts. **c** Heatmap of mRNA with sex differences in expression in X^wt^/X^wt^, X^ogt−^/X^wt^, X^wt^/Y trophoblasts. Expression patterns of trophoblast genes (columns) with sex differences are mimicked by *Ogt* reduction in female trophoblasts (individual placentas = rows). *Z*-scores plotted across individuals for each gene. **d** Venn diagram displaying the number of differentially expressed genes in X^wt^/Y vs. X^wt^/X^wt^ (*n* = 4560 genes), X^ogt−^/X^wt^ vs. X^wt^/Y (*n* = 251 genes), and X^ogt−^/X^wt^ vs. X^wt^/X^wt^ (*n* = 2197 genes) trophoblasts and the number of differentially expressed genes common in these groups. **e** A total of 1094 of the 2609 trophoblast genes enriched in X^wt^/X^wt^ relative to X^wt^/Y were significantly decreased by trophoblast-specific *Ogt* reduction (X^ogt−^/X^wt^). **f** A total of 432 trophoblast genes with increased relative expression in X^wt^/Y were enhanced in X^ogt−^/X^wt^ relative to X^wt^/X^wt^. **g** Only 7 of the 2609 genes with X^wt^/X^wt^-biased sex differences in expression were upregulated by trophoblast-specific *Ogt* reduction (X^ogt−^/X^wt^). **h** A total of 4 of the 1951 X^wt^/Y-biased genes with basal sex differences were downregulated in X^ogt−^/X^wt^ trophoblasts. *N* = 3 X^wt^/Y, *n* = 4 X^wt^/X^wt^, and *n* = 5 X^ogt−^/X^wt^ placentas, from five individual litters with *n* = 1/litter/group to control for litter effects

Using RNA-Sequencing and RiboTag technology^28^ to isolate trophoblast cell-specific, actively translated mRNAs, we identified 4560 genes with significant sex differences in expression at embryonic day (E)12.5 (Fig. 1a–d), far more than previous analyses using whole placenta containing heterogeneous and maternal cell populations^24,29–32^. The large number of sex differences in this fetally derived placental cell type demonstrates their potential importance for establishing sex differences in trans-placental signals and fetal development. Functional gene ontology (GO) analysis revealed only 2 biological processes enriched in male trophoblasts relative to females, whereas 81 biological processes were statistically enriched in female trophoblasts relative to males (Supplementary Figure 1a-d; Supplementary Data File 1). This finding highlights the presence of sex specificity in trophoblast function and suggests underlying sex-specific epigenetic programs in placental trophoblasts, which might promote the disparity in developmental vulnerabilities between sexes.

Consonant with this idea and with the established role for OGT in regulating epigenetic processes controlling large gene sets, we found that genetic reduction of *Ogt* specifically in female trophoblasts (X^ogt−/^X^wt^, Supplementary Figure 2) recapitulated patterns of male (X^wt^/Y) trophoblast gene expression compared to wild-type females (X^wt^/X^wt^; Fig. 1a, c, d) and eliminated many sex differences in trophoblast gene expression compared to wild-type males (X^wt^/Y; Fig. 1b, c, d). There were 2197 changes in gene expression resulting from trophoblast-specific *Ogt* reduction in the female placenta, 1534 of which were genes originally identified with significant sex differences (Fig. 1c, d), illustrating the importance of *Ogt* levels in determining sex differences in trophoblast gene expression patterns. Of the 2609 genes elevated in X^wt^/X^wt^ trophoblasts relative to X^wt^/Y, 1094 were decreased (i.e., masculinized) by trophoblast *Ogt* reduction (Fig. 1e; Supplementary Data File 1). Similarly, of the 1951 genes with decreased relative expression in X^wt^/X^wt^ trophoblasts versus X^wt^/Y, 432 were increased in X^ogt−/^X^wt^ trophoblast (Fig. 1f; Supplementary Data File 2). Interestingly, we found that many of the same GO biological processes were assigned to gene sets increased in X^wt^/X^wt^ compared to X^ogt−/^X^wt^ and X^wt^/Y trophoblasts (Supplementary Figure 1; Supplementary Data File 2), demonstrating the functional relationship between placental *Ogt* dosage and sex differences in trophoblast function.

### Placental *Ogt* regulates sex differences in the hypothalamus

**Fig. 2:**
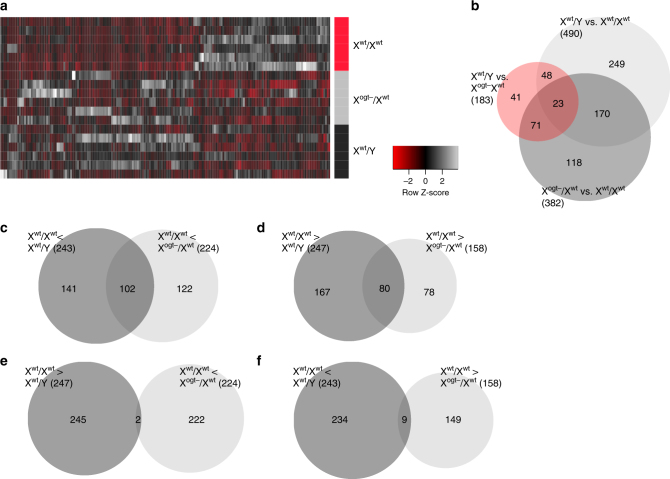
Gene expression patterns are masculinized in the E18.5 female hypothalamus by placental *Ogt* reduction. **a** Heatmap of mRNAs with sex differences in hypothalamic expression (490 genes, FDR adjusted *p* < 0.05) in trophoblast X^wt^/X^wt^, X^ogt−^/X^wt^, X^wt^/Y at E18.5. Expression patterns of hypothalmic genes (columns) with sex differences were mimicked by *Ogt* reduction in female trophoblasts (individual placentas = rows). *Z*-scores plotted across individuals for each gene. **b** Venn diagram displaying the number of differentially expressed genes in the hypothalamus of animals with X^wt^/Y vs. X^wt^/X^wt^ (*n* = 490 genes), X^ogt−^/X^wt^ vs. X^wt^/Y (*n* = 182 genes), and X^ogt−^/X^wt^ vs. X^wt^/X^wt^ (*n* = 381 genes) trophoblasts and the number of differentially expressed genes common in these groups. **c**–**f** Venn diagrams displaying overlapping directional changes in hypothalamic gene expression in trophoblast X^wt^/X^wt^, X^ogt−^/X^wt^, X^wt^/Y fetuses. **c** A total of 102 genes enriched in the X^wt^/Y male hypothalamus relative to X^wt^/X^wt^ females were also enriched in the hypothalamus of X^ogt−^/X^wt^ females. **d** Similarly, 80 genes enriched in the X^wt^/X^wt^ females hypothalamus relative to X^ogt−^/X^wt^ females were also enriched in the hypothalamus of X^wt^/X^wt^ females compared to X^wt^/Y males. **e** Only 2 genes with higher hypothalamic expression in X^ogt−^/X^wt^ females relative to X^wt^/X^wt^ females were higher in X^wt^/X^wt^ females relative to X^wt^/Y males. **f** Similarly, only 9 genes with higher hypothalamic expression in X^wt^/X^wt^ females relative to X^ogt−^/X^wt^ females were higher in X^wt^/Y males relative to X^wt^/X^wt^ females. *N* = 6/group from 7 litters with a maximum of 2/group/litter used to control for litter effects

In our previous studies, genetic placental *Ogt* reduction recapitulated the HPA stress axis dysregulation and metabolic dysfunction characteristic of male offspring exposed to prenatal stress^26^. Therefore, we predicted that sex differences in trophoblast *Ogt* contribute to sex differences in neurodevelopment. Specifically, we focused on the hypothalamus, a neuroendocrine brain region critical for stress and metabolic homeostasis, which is sensitive to prenatal insults and frequently altered in patients with neurodevelopmental disorders^7,33^. We hypothesized that placental OGT, through its transcriptional control and downstream trans-placental signals, would influence key known robust sex differences in hypothalamic development. To avoid confounds associated with postnatal environmental influences, we performed RNA-Sequencing on hypothalamic mRNA at E18.5, a late gestational time point where the hypothalamus is largely differentiated, from trophoblast X^wt^/X^wt^, X^wt^/Y, and X^ogt−^/X^wt^ fetuses. We found that more than one-third, 193 of 490, of the genes with significant sex differences were altered in the female hypothalamus when placental *Ogt* was reduced (Fig. 2a, b), 182 of which statistically switched to a male expression pattern (Supplementary Data File 3), a confirmation of the critical importance of placental *Ogt* levels and corresponding trans-placental signals in sex-specific neurodevelopment.

Whereas, 382 genes were altered in the female hypothalamus by placental *Ogt* reduction (X^wt^/X^wt^ vs. X^ogt−^/X^wt^), only 183 genes were differentially expressed between males and placental *Ogt* hemizygous females (X^wt^/Y vs. X^ogt−^/X^wt^; Fig. 2b). Further, the directionality of changes in differentially expressed hypothalamic genes in trophoblast X^wt^/X^wt^ vs. X^ogt−^/X^wt^ resembled the directionality of differences in placental X^wt^/X^wt^ vs. X^wt^/Y (Supplementary Data File 3). Expression patterns of 182 hypothalamic genes were similar among trophoblast X^ogt−/^X^wt^ females and X^wt^/Y males relative to X^wt^/X^wt^ females, with the expression of 102 genes increased in trophoblast X^ogt−/^X^wt^ and X^wt^/Y males relative to X^wt^/X^wt^ females (Fig. 2c) and 80 genes decreased relative to X^wt^/X^wt^ females (Fig. 2d), including genes involved in sexual differentiation of the hypothalamus (*Esr1, Cyp19a1*), genes linked to metabolic and stress responsivity (*Pmch, Npy2r, Grp*), and those previously implicated in neurodevelopmental disorders (*Foxp2, Foxg1, Oxt, Grp, Chrna4*)^34^. Only 2 of the 247 genes upregulated in X^wt^/X^wt^ vs. X^wt^/Y were increased in trophoblast X^ogt−^/X^wt^ hypothalamus relative to X^wt^/X^wt^ (Fig. 2e), and a mere 9 hypothalamic genes downregulated in X^wt^/X^wt^ vs. X^wt^/Y were commonly reduced in placental X^ogt−^/X^wt^ vs. X^wt^/X^wt^ (Fig. 2f). The marked masculinization of hypothalamic gene expression patterns resulting from trophoblast-specific *Ogt* reduction in females confirms the importance placental OGT as a mediator of sex-specific neurodevelopment, and may underlie sex differences in long-term programmatic outcomes following prenatal perturbations.

### OGT establishes sex differences in placental H3K27me3

**Fig. 3:**
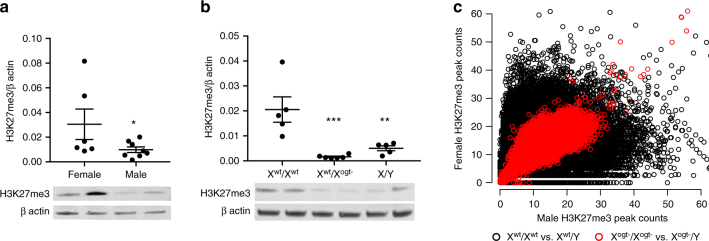
OGT regulates placental H3K27me3 levels. **a** Western immunoblot quantification of H3K27me3 in human term placenta (*t*-test, *t*_(12)_ = 1.896, *p* = 0.0412, *n* = 6 female, *n* = 8 male). **b** This sex difference was also present in E12.5 mouse placenta and was eliminated by placental *Ogt* reduction in female trophoblasts, X^ogt−^/X^wt^ (ANOVA, *F*(2,13) = 12.9, *p* = 0.0008, *n* = 5 X^wt^/X^wt^, X^wt^/Y, *n* = 6 X^ogt−^/X^wt^ from 10 litters with *n* = 1/group/litter). **c** Scatterplot of ChIP-Seq H3K27me3 peak counts within 2 Kb of transcription start sites showing widespread sex differences (black circles) were greatly reduced by trophoblast-specific *Ogt* deletion in both sexes (red circles; *n* = 3 X^wt^/X^wt^, *n* = 4 X^wt^/Y, X^ogt−^/X^ogt−^ & X^ogt−^/Y from 8 litters with *n* = 1/group/litter). *p < 0.05 compared to X^wt^/X^wt^. Bars represent mean ± sem

*OGT*-facilitated H3K27me3 patterning is a plausible mechanism enabling its ability to establish large-scale sex differences in trophoblast gene expression governing prenatal susceptibility. More stringent epigenetic repression of chromatin state via high levels of H3K27me3 may restrict transcriptional fluctuations in response to insults, providing females with in utero resiliency relative to males. Accordingly, we hypothesized that placental OGT acts as a mediator of sex-specific placental gene expression through its regulation of H3K27me3. We identified a female-biased sex difference in H3K27me3 in both human (Fig. 3a) and E12.5 mouse placentas (Fig. 3b). While no longer statistically significant, this pattern of enriched H3K27me3 in the female mouse placenta persisted through late gestation (Supplementary Figure 3a). Consistent with OGT’s previously described role in EZH2 stabilization and activation^27^, we found that the sex difference in placental H3K27me3 was *Ogt* -dependent, as genetic reduction of *Ogt* masculinized female placental H3K27me3 levels (Fig. 3b). However, this *Ogt* reduction effect on H3K27me3 appeared specific as it did not promote pervasive changes in other histone post-translational modifications in the placenta (Supplementary Figure 3b, c). Further, chromatin immunoprecipitation (ChIP)-Sequencing of H3K27me3 in the E12.5 placenta revealed marked sex differences in H3K27me3 patterning across the entire genome (Fig. 3c). Trophoblast-specific *Ogt* reduction eliminated genome-wide sex differences in H3K27me3 peaks (±2 Kb from transcription start sites), demonstrating a critical OGT function in establishing sex-specific H3K27me3 configurations in placental trophoblasts.

### Trophoblast-specific *Ezh2* reduction promotes vulnerability

**Fig. 4:**
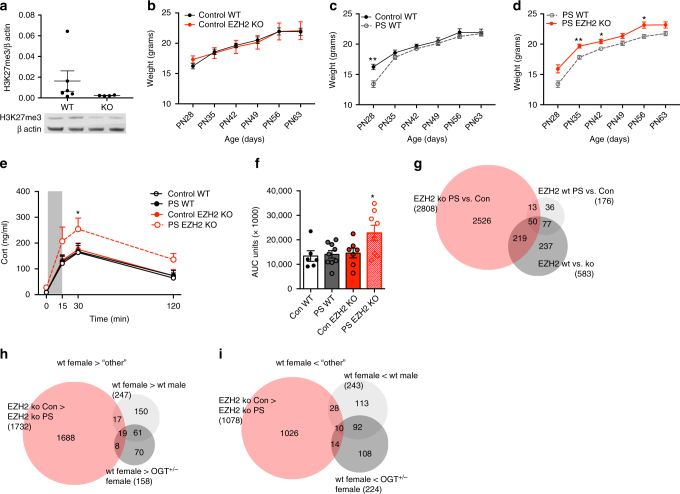
Placental *Ezh2* genetic deletion promotes female vulnerability to prenatal stress. **a** Trophoblast-specific *Ezh2* KO patterns H3K27me3 in female E12.5 placentas (*t*-test, *t*_(8)_ = 1.142, *p* = 0.1433; *n* = 6 WT, *n* = 4 KO from 6 litters with *n* = 1/group/litter). **b** Trophoblast-specific *Ezh2* KO alone has no impact on post-weaning body weight in females unexposed to prenatal stress. **c** Although prenatal stress (PS) exposed WT females weigh less at weaning (PN28, *t*(13) = 3.989, *p* = 0.009), there were no long-term effects of stress on WT female body weights. **d** However, when females with trophoblast-specific *Ezh2* KO were exposed to PS they showed increased body weights into adulthood (PN28, *t*(9) = 2.846, *p* = 0.056; PN35, *t*(16) = 3.987, *p* = 0.006; PN42, *t*(16) = 2.857, *p* = 0.044; PN56, *t*(16) = 3.258, *p* = 0.024; PN63, *t*(16) = 2.346, *p* = 0.063; *n* = 8 Con WT, *n* = 10 PS WT, *n* = 4 Con *Ezh2* KO, *n* = 8 PS *Ezh2* KO from 7 PS and 8 Con litters with a maximum of 2/genotype/litter). **e** Plasma corticosterone levels were significantly higher in response to a 15-min restraint stress in females with trophoblast- *Ezh2* KO exposed to PS relative to control WT and PS WT groups (Two-way repeated measures ANOVA, *F*_int_(9,78) = 0.4101, *p* = 0.9261, *F*_time_(3,78) = 38.66, *p* < 0.0001, *F*_group_(3,26) = 3.76, *p* = 0.0229, *F*_subjects(matching)_(26,78) = 1.913, *p* = 0.0235). **f** Corticosterone area under the curve (AUC; ANOVA, *F*(3,26) = 3.77, *p* = 0.023; *n* = 6 Con WT, *n* = 9 PS WT, *n* = 7 Con *Ezh2* KO, *n* = 8 PS *Ezh2* KO from 7 PS and 8 Con litters with a maximum of 2/genotype/litter). **g** Venn diagram displaying the number of differentially expressed genes in the hypothalamus of trophoblast WT female fetuses exposed to PS (*n* = 180 genes), non-stress exposed trophoblast *Ezh2* WT vs. KO females (*n* = 603 genes), and females with trophoblast-specific *Ezh2* KO exposed to PS vs. control treatment (*n* = 2884 genes) at E18.5 (*n* = 6 Con WT, *n* = 5 PS WT, *n* = 3 Con *Ezh2* KO, *n* = 4 PS *Ezh2* KO from 14 PS and 14 Con litters with a maximum of 1/genotype/litter). **h** Venn diagram comparing enriched gene expression in the WT female hypothalamus compared to males, trophoblast X^ogt−^/X^wt^ females, and trophoblast *Ezh2* KO females. **i** Venn diagram comparing downregulated hypothalamic genes in WT females relative to males, trophoblast X^ogt−^/X^wt^ females, and trophoblast *Ezh2* KO females. *p < 0.05, **p < 0.01. Bars represent mean ± sem

OGT regulates a multitude of cellular processes^25^. Therefore to establish the necessity of high levels of H3K27me3 in female resilience to prenatal stress, we genetically reduced *Ezh2* in trophoblasts (Supplementary Figure 4) to decrease H3K27me3 levels in the female placenta (Fig. 4a). This manipulation did not disrupt other critical histone post-translational modifications in the placenta (Supplementary Figure 3d, e). Many neurodevelopmental disorders likely arise as the result of gene X environment interactions^7^, in which detrimental outcomes are produced by the interaction of genetic susceptibilities and environmental events which alone are innocuous. We hypothesized that experimentally induced female genetic vulnerability (i.e., reduced H3K27me3) would interact with an environmental insult (prenatal stress) to produce the metabolic and HPA stress axis dysfunction typical in male offspring following prenatal stress exposure. Trophoblast-specific knockout (KO) of *Ezh2* alone had no effect on female body weight gain or HPA axis sensitivity (Fig. 4b, e, f). As previously reported^22,35,36^, wild-type (WT) female offspring exposed in utero to prenatal stress showed little change in post-weaning body weight gain (Fig. 4c) or HPA stress axis function in adulthood (Fig. 4e, f). However, as we predicted, the combination of trophoblast *Ezh2* reduction × prenatal stress produced the expected susceptibility to neuroendocrine dysregulation. Females with trophoblast *Ezh2* reduction exposed to prenatal stress showed lasting increases in post-weaning body weight relative to WT females (Fig. 4d), and showed a hyper-reactive corticosterone response to a restraint stressor (Fig. 4e, f), suggesting long term metabolic and HPA stress axis dysfunction. As previous reported in this phenotype, we found no effects of genotype or prenatal stress exposure on behavior in an open field or elevated plus maze (Supplementary Figure 5), suggesting that female body weight changes are not due to locomotor differences.

The marked metabolic and HPA stress axis dysregulation exhibited by females with trophoblast-specific *Ezh2* reduction exposed to prenatal stress implies a change in developmental programming of the hypothalamus. RNA-sequencing of the E18.5 hypothalamus from trophoblast-specific *Ezh2* KO mice revealed extensive disruptions of gene expression patterns, specifically in females exposed to prenatal stress compared to control females (Fig. 4g), suggesting that the developmental impact of environmental insults synergize with placental genotype and epigenetic status to direct trans-placental signals important for hypothalamic development. Statistically enriched amongst the hypothalamic genes were notable chromatin modifiers, including *Setd7, Tet1, Kmt2a, Ep300, Nipbl, Sirt1, Ogt, Kat2b, Kdm3a, Kdm5a*, and *Ezh2* (Supplementary Data File 4), implicating trans-placental signals as a mechanism for long-term epigenetic programming of hypothalamic function. Although, we found substantial overlap in hypothalamic genes enriched (Fig. 4h) and decreased (Fig. 4i) in trophoblast WT females relative to both males and trophoblast X^ogt−^/X^wt^ females, there were far fewer hypothalamic genes that shared predictable expression patterns in the hypothalamus of females with trophoblast-specific *Ezh2* reduction exposed to prenatal stress. While many genes were significantly altered in the hypothalamus of placental *Ezh2* KO females exposed to prenatal stress relative to controls, suggesting a role of placental function in regulation of their expression, the directionality of these changes was not always consistent with our hypothesis, such that 10 of the 102 genes that increased in expression in males and trophoblast X^ogt−^/X^wt^ females relative to WT females were increased in placental *Ezh2* KO females (Fig. 4i; *p* = 9.457e−11, Fisher’s exact test (FET) for enrichment of overlapping upregulated genes in *Ezh2* KO PS females vs. *Ezh2* KO control females and WT males vs. WT females), and 19 of the 80 downregulated genes was lower in placental *Ezh2* KO females (Fig. 4h; Supplementary Data File 5; *p* = 2.118e−15, FET for enrichment of overlapping downregulated genes in *Ezh2* KO PS females vs. *Ezh2* KO control females and WT males vs. WT females), suggesting additional mechanisms involved in these complex cellular processes and trans-placental signals.

## Discussion

While sex differences in prenatal sensitivity to environmental perturbations are well documented, a paucity of molecular mechanisms responsible for imparting female resilience and male risk during this critical period in neurodevelopment have been reported. Here we provide evidence that the X-linked metabolic and epigenetic enzyme, OGT, serves as a shield to gestational perturbations in females, in part through its regulation of the broad transcriptional repressive mark, H3K27me3, and transcriptomic placental programs. Our finding that female-biased trophoblast genes were easily sorted into gene sets associated with GO biological processes but male-biased genes were not, may suggest that female trophoblast gene expression, at baseline, is epigenetically organized differently than male trophoblast gene expression. This finding corresponds with our data that males and female trophoblasts differ in their epigenomic programming due to differences in placental *Ogt* dosage and may indicate that placental OGT-mediated epigenomic programing promotes greater baseline homogeneity in female trophoblast gene expression relative to males. When repressive transcriptional regulation is reduced in females, via *Ogt* or *Ezh2* manipulation, male-like transcriptional variability is induced, possibly underlying environmental risk for neurodevelopmental vulnerability.

Large *Ogt*-driven differences in gene expression demonstrate the importance of sex chromosome complement for trophoblast cell function and support the placenta’s powerful role in introducing sex differences in fetal development. Our data show that epigenetic and transcriptional patterns in the placenta contribute to programming sex differences in the developing hypothalamus, a region commonly disrupted in neurodevelopmental disorders, including autism, ADHD, and schizophrenia, which is a critical integrator of environmental information and orchestrator of organismal homeostasis^33,37–41^. Accordingly, placental H3K27me3 determines female resilience to the metabolic and stress axis dysfunction produced by prenatal stress exposure, a phenotype that we have previously shown to be male specific. Our previous studies had revealed that trophoblast-specific *Ogt* reduction recapitulated the male-specific phenotype. However, the broad transcriptional control placental OGT played in regulation of sex differences in the placenta, as well as in impacting trans-placental signals necessary for similar sex differences in hypothalamic gene expression was previously unknown. Our current results mechanistically demonstrate the involvement of placental epigenetic signaling in neurodevelopment.

Here we focus on a single mode by which OGT mediates in transcriptional epigenetic programming underlying neurodevelopmental resilience: by determining H3K27me3 levels. Although OGT directly influences EZH2 in breast cancer cells^27^ and trophoblast-specific *Ezh2* reduction in conjunction with prenatal stress exposure recapitulated key aspects of the male-biased phenotype typical of prenatal stress exposed mice, the hypothalamic gene expression profiles in *Ezh2* KO females exposed to prenatal stress showed little overlap with those in *Ogt* hemizygous female hypothalami, which likely suggests that mediation of H3K27me3 levels is one of several mechanisms whereby placental OGT influences brain development. OGT’s capacity to alter numerous cellular signaling events, epigenomic programming, and transcriptional activation could mean that regulation of H3K27me3 is secondary or tertiary to an upstream modification made by OGT. For instance, in addition to potentially impacting trophoblast gene expression profiles via direct activation of RNA polymerase II^42^, OGT can directly modify core histones^43–45^ and has been reported to influence several other important epigenetic modifications that could lead to sex differences in trophoblast gene expression to ultimately influence brain development and vulnerability. In addition, OGT can associate with TET2/3, members of the ten eleven translocation family of 5-methylcytosine oxidizing enzymes that enable DNA demethylation^46–49^. OGT-TET2/3 containing complexes have been shown to promote the canonically permissive epigenetic modification, H3K4me3^50^. Thus, sex differences in OGT likely program transcriptional and epigenetic landscapes via several different mechanisms.

As *Ogt* is an X-linked gene and *Ogt* levels are higher in female trophoblast cells, it is likely that *Ogt* escapes X-inactivation here. Although, we did not directly assess this in our model, previous studies have reported that *Ogt* as an X-inactivation escapee in extra-embryonic tissues^51–54^, providing female trophoblasts with two active copies versus a single copy in males. H3K27me3 is a critical mediator of X-inactivation, and thus is expected to be elevated in XX female cells. While *Ogt* reduction diminished H3K27me3 levels in trophoblasts, we identified only 116 annotatable X-linked genes that were also altered in these cells, 88 of which showed enhanced expression when H3K27me3 was reduced, suggesting that we did not impact widespread release of X-inactivation (Supplementary Data File 6). This is not necessarily surprising considering the large number of coordinated epigenetic modifications (histone modifications, DNA methylation, and long-non-coding RNAs) necessary to silence the inactive X^55,56^ and that our transgenic modification of *Ogt* also did not produce vast alterations to histone modifications other than H3K27me3 as previously reported^27^ (Supplementary Figure 3).

Although additional studies are needed to further define how placental OGT impacts the epigenomic landscape responsible for healthy in utero development, our findings provide evidence that its regulation of H3K27me3 is critical in determination of trans-placental signals involved in brain programming. These outcomes validate the importance of the placenta as an active intercessor between the maternal milieu and fetal development, and demonstrate that sex differences in placental genetic and epigenetic patterning are critical in this process. Identifying the trans-placental signals that determine neurodevelopmental vulnerability and retrospective studies examining gene × environment interactions utilizing human extra-embryonic tissues will bring us even closer to understanding how male-biased neurodevelopmental diseases arise.

## Methods

### Animals

Mice were maintained on a 12:12 light/dark cycle with ad libitum access to food and water. For trophoblast-specific reduction of *Ogt*, trophoblast Cre (P.Cre)^57^ positive females [B6.129-CYP19-Cre, P.Cre^+^/^−^)] were crossed with P.Cre negative, OGT floxed males [B6.129-Ogttm1Gwh/J, X^Ogt−^/ Y], resulting in offspring representing all potential genotypes. For RiboTag breedings, RiboTag heterozygous [B6.129-Rpl22-tm1.1Psam (RiboTag^+^/^−^)/placenta Cre positive (P.Cre^+^/^−^)] females were bred to [*Ogt* floxed B6.129-Ogttm1Gwh/J (X^Ogt−^/ Y)/RiboTag heterozygous Rpl22-tm1.1Psam (RiboTag^+^/^−^)/ placental Cre negative (P.Cre^−^/^+^)] males. For EZH2 breedings, B6.129-Ezh2^tm2Sho^/J^+/+^ females were crossed with male B6.129-Ezh2^tm2Sho^/J^+/−^/ P.Cre^+^/^−^. Mice were mated and checked daily at 7:00 AM (lights on) for the presence of a copulation plug. Noon on the day that the plug was observed was considered to be embryonic day 0.5 (E0.5). Prenatal stress was performed as previously described^22^. Briefly, dams were randomly assigned to treatment group and exposed to mild chronic variable stress from gestational day 1–7. Each mouse was exposed to 1 stressor per day. Stressors included overnight light, overnight noise, overnight wet bedding, 15 min restraint stress, 1 h exposure to predator odor, multiple cage change, and novel objects (marbles) placed in cage overnight. All animal protocols were reviewed and approved by the Institutional Animal Care and Use Committee of the University of Pennsylvania. Sample sizes for all experiments were based on previous expertize with these methods.

### Animal weights

For studies in which animals were raised to adulthood, mice were weaned at PN28, given ear tags for individual identification and weighed weekly until 2 weeks prior to HPA stress axis testing. Because not all animals were weighed at weaning, repeated measures ANOVAs could not be completed on our full weight dataset. Instead, *t*-tests were conducted at each time point with Holm-Sidak multiple comparison correction with *α* = 0.05 using Prism (GraphPad).

### Tissue collection

Human placental tissue from de-identified neonates was quartered by cutting traverse sections through the location of the emergence of the umbilical cord in two directions. A total of 5 mg biopsies were obtained from the fetal side of the placenta, 5 cm from the umbilical cord. Biopsies were flash frozen in liquid nitrogen and stored at −80 °C until use. Parental consent was given for use of placental samples. Human tissue collection protocols were approved by the Institutional Review Board of the University of Pennsylvania. Mouse placentas and embryonic somatic tissue were dissected at E12.5 and E18.5. DNA was extracted from the embryonic somatic tissue for genotyping and sexing. One-2 male and female placenta per genotype from each litter was used for each analysis to eliminate potentially confounding litter effects; fetal heads were collected whole and placentas were halved, flash-frozen in liquid nitrogen and stored at −80 °C until processed as below. For E18.5 hypothalamic RNA-Seq RNA collection, fetal heads were sectioned at 300 μm on a Cryostat and a 1 mm tissue punch was collected in Buffer RLT (RNeasy Micro Kit; Qiagen) and RNA was immediately isolated. All tissue was deindentified and coded such that experiments were blind to genotype/treatment.

### Nuclear extraction and western immunoblot

For histone westerns, halved E12.5 and E18.5 mouse placentas and 2 cm^2^ human placenta biopsies were subject to nuclear extraction using established protocols. Briefly, placentas were homogenized with a pestle in sterile PBS on ice, homogenates were centrifuged at 1200×*g* for 10 min at 4 °C, pellets were washed with PBS, and resuspended in 800 μl of Buffer A (10 mM HEPES, pH 7.8, 10 mM MgCl_2_, 0.1 mM EDTA, 1 mM DTT, protease inhibitor cocktail (Sigma)). Following a 15 min incubation on ice, 0.05% NP-40 was added, samples vigorously vortexed, and nuclear extracts pelleted at 14,000×*g* for 30 s. Nuclear pellets were resuspended in Buffer B (50 mM HEPES, pH 7.8, 50 mM KCl, 300 mM NaCl, 0.1 mM EDTA, 1 mM DTT, protease inhibitor cocktail) and subject to Bradford assay for protein quantification. For OGT, O-glycNAc, and EZH2 westerns, halved E12.5 placentas and brains were homogenized in RIPA buffer (Sigma) with protease inhibitor cocktail (Sigma), centrifuged at 16000×*g* for 20 min at 4 °C, and whole-cell lysate protein concentration in supernatant was quantified via Bradford assay. A total of 30 μg of placental protein was loaded per lane for gel electrophoresis onto a NuPAGE 10% Bis-Tris gel (Life Technologies). After running and transfer of proteins to a nitrocellulose membrane (Life Technologies), membranes were blocked with Odyssey blocking buffer (Li-Cor) and probed with rabbit anti-H3K27me3 (1:1000; Millipore 07–449), rabbit anti-H3K4me3 (1:1000, abcam ab8580), rabbit anti-H3K9ac (1:1000, Cell Signaling C5B11), rabbit anti-OGT (1:1000, Sigma DM-17), mouse anti-O-glycNac (1:1000, Biolegend CTD110.6), rabbit anti-EZH2 (1:2500; Millipore 07–689), and/or mouse anti-beta actin (1:30,000; Sigma A5441) followed by incubation in IRDye800-conjugated donkey anti-rabbit secondary (1:20,000; Li-Cor) and or IRDye680-conjugated goat anti-mouse secondary (1:20,000; Li-Cor). We used single-tailed *t*-tests for most two-group analyses because we had a priori hypotheses about the directionality of the changes in protein expression based on our previous data on sex differences and OGT regulation of H3K27me3 and predicted directional effects of *Ezh2* and *Ogt* transgenic manipulation. As we did not have a priori hypotheses about the directionality of H3K4me3 or H3K9ac, we used two-tailed *t*-tests for these analyses. Western blot quantification of H3K27me3 in male, female, and trophoblast X^ogt−^/X^wt^ by one-way ANOVA with *α* = 0.05 using Prism (GraphPad). Images of full western blots are shown in Supplementary Figure 6.

### Chromatin immunoprecipitation sequencing (ChIP-Seq)

Placental chromatin isolation and sample preparation was performed as described previously^26^. Briefly, chromatin was isolated from halved placentas following six 5 min rounds on sonication on ice, using Dynabeads Protein G (Life Tech) and an H3K27me3 antibody (Millipore 07–449; 1:15). Multiplexed libraries were prepared from 10 ng of chromatin using the Illumina TruSeq ChIP Sample Preparation Kit. Libraries were sequenced (50-bp single end) on an Illuminia NextSeq500; fastq files were aligned to the genome using Bowtie2^58^, enriched peaks relative to input for H3K27me3 were calculated using HOMER^59^, and these peak counts were then used for subsequent analyses in DESeq^60^.

### RiboTag mRNA immunoprecipitation

Actively translated mRNA was immunoprecipitated from placental trophoblasts at E12.5 using RiboTag mice as previously described^28^. Briefly, whole placentas were dounce homogenized in 3 ml supplemented homogenization buffer (50 mM Tris, pH 7.5, 100 mM KCl, 12 mM MgCl_2_, 1% NP-40, 1 mM DTT, 200 U/mL RNasin (Promega), 1 mg/mL heparin, 100 μg/mL cyclohexamide, protease inhibitor cocktail (Sigma)). Samples were centrifuged at 10,000×*g* for 10 min at 4 °C, 800 μl of supernatant was added incubated with 5 μl of anti-HA.11 clone 16B12 antibody (BioLegend) for 4 h at 4 °C. A total of 400 μl of Dynabeads Protein G (Life Tech) were washed with supplemented homogenization buffer and incubated with supernatant-antibody complex overnight at 4 °C. The following day, bead-antibody-protein complexes were washed three times for 5 min with high-salt buffer (50 mM Tris, pH 7.5, 300 mM KCl, 12 mM MgCl_2_, 1% NP-40, 1 mM DTT, 100 μg/mL cyclohexamide. Immediately following washes, Qiagen Buffer RLT was added to complexes and RNeasy protocol was followed per the manufacturer’s instructions to isolate RNA from complexes.

### RNA sequencing and analysis

RNA from placental RiboTag and hypothalamic punches was quantified on a NanoDrop 2000 (Thermo Scientific) spectrophotometer and quality was assessed on a BioAnalyzer (Agilent). RNA-Seq libraries were constructed using a TruSeq Stranded mRNA Sample Preparation Kit using the low sample protocol on 350 ng total RNA from E18.5 hypothalamic punches and 700 ng RiboTag isolated mRNA from E12.5 placenta. Libraries sizes and concentrations were confirmed on a TapeStation 4200 (Agilent) and by KAPA assay (KAPA Biosystems). Individually barcoded libraries were pooled and sequenced on an Illuminia NextSeq500 (75-bp single end). Alignment and read counting was performed in RSubread using featureCounts^61,62^. Library size normalization and differential expression analysis on normalized read counts was performed using DESeq^60^. Differential expression was based on an FDR adjusted *p*-value <0.05, with no fold change cutoff to maximize the number of genes included in our comparisons of placental and hypothalamic masculinization. All statistical analyses were performed in and figures generated in R on normalized counts^63^ (version 3.1.1) using base packages and plyr^64^, gplots^65^, RColorBrewer^66^, and venneuler^67^. Database for Annotation, Visualization and Integrated Discovery (DAVID) functional annotation clustering^68^ was used for the gene ontology (GO) pathway analysis on differentially expressed gene lists.

### Corticosterone response to restraint stress

Naive 11–12-week-old-female mice were restrained in a 50 ml conical tube for 15 min beginning at time 0 min and were immediately returned to their home cage at the conclusion of restraint (time 15 min). A single-tail snip was made at time 0 min, removing <1 mm tissue from the distal tip. A total of 10 μL of tail blood was collected by micropipette at 0, 15, 30, and 120 min into EDTA-treated tubes. Corticosterone levels were quantified by radioimmunoassay (MP Biomedicals) in 3 μL plasma. All testing was completed 2–5 h after lights on. Cort rise and fall data were analyzed by repeated measures two-way ANOVA and total Cort (area under curve; AUC) was analyzed by one-way ANOVA with *α* = 0.05 using Prism (GraphPad). Samples that 2 standard deviations above or below the mean were treated as outliers and excluded.

### Behavioral testing

Two weeks following HPA stress axis assessment, mice were subject to testing in an open field during the dark cycle to determine the impact of trophoblast-specific EZH2 reduction on locomotor activity and anxiety-like behavior. The open field apparatus is a white Plexiglas box 50 × 50 × 22 cm with a 16 12 × 12 cm grid drawn on the floor. Testing was conducted with 120 lux in the center of the box, 2–4 h after lights out (9:00 to 11:00 PM). Each mouse was placed in the center of the box to initiate the 10 min test and tracked using AnyMaze (Stoelting) software. Two weeks following completion of open field testing, mice were subject to locomotor and anxiety-like behavior testing on an elevated plus maze during lights on. Testing duration was 5 min with a light intensity of 6 lux in the open arms. AnyMaze software was used to measure distance, grid crosses and time in open arms. Mice that jumped off open arms during testing were excluded from analysis. Behavioral data collected by an experimenter blind to treatment and analyzed by two-way ANOVA (genotype × treatment) using Prism software (Graphpad).

### Data availability

Alignment files for RNA sequencing studies are available in NCBI’s Sequence Read Archive (SRA) under study accession #SRP144932. Due to hardware failure, raw data files for H3K27me3 ChIP-Seq are not available. Downstream ChIp-Seq analysis files generated in Homer are available in Dryad. Additional data are available upon reasonable request.

## Electronic supplementary material

Supplementary Information

Description of Additional Supplementary Files

Supplementary Data 1

Supplementary Data 2

Supplementary Data 3

Supplementary Data 4

Supplementary Data 5

Supplementary Data 6

## References

[1] Regitz-Zagrosek V (2012). Sex and gender differences in health: Science & Society Series on Sex and Science. EMBO Rep..

[2] Castle DJ, Murray RM (1991). The neurodevelopmental basis of sex differences in schizophrenia. Psychol. Med..

[3] Baron-Cohen S, Knickmeyer RC, Belmonte MK (2005). Sex differences in the brain: implications for explaining autism. Science.

[4] Aleman A, Kahn RS, Selten JP (2003). Sex differences in the risk of schizophrenia: evidence from meta-analysis. Arch. Gen. Psychiatry.

[5] Tyson JE, Parikh NA, Langer J, Green C, Higgins RD (2008). Intensive care for extreme prematurity—moving beyond gestational age. N. Engl. J. Med..

[6] Drevenstedt GL, Crimmins EM, Vasunilashorn S, Finch CE (2008). The rise and fall of excess male infant mortality. Proc. Natl Acad. Sci. USA.

[7] Bale TL (2010). Early life programming and neurodevelopmental disorders. Biol. Psychiatry.

[8] Nugent BM, Bale TL (2015). The omniscient placenta: metabolic and epigenetic regulation of fetal programming. Front. Neuroendocrinol..

[9] Rossant J, Cross JC (2001). Placental development: lessons from mouse mutants. Nat. Rev. Genet..

[10] Amankwah KS, Kaufmann RC (1984). Ultrastructure of human placenta: effects of maternal drinking. Gynecol. Obstet. Invest..

[11] Eguchi Y (1989). Histological changes in the placenta induced by maternal alcohol consumption in the rat. Neonatology.

[12] Ganapathy V (2011). Drugs of abuse and human placenta. Life Sci..

[13] Gheorghe CP, Goyal R, Mittal A, Longo LD (2010). Gene expression in the placenta: maternal stress and epigenetic responses. Int. J. Dev. Biol..

[14] Godfrey KM, Barker DJP (1995). Maternal nutrition in relation to fetal and placental growth. Eur. J. Obstet. Gynecol. Reprod. Biol..

[15] Godfrey K, Robinson S, Barker DJP, Osmond C, Cox V (1996). Maternal nutrition in early and late pregnancy in relation to placental and fetal growth. BMJ.

[16] Jauniaux E, Burton GJ (2007). Morphological and biological effects of maternal exposure to tobacco smoke on the feto-placental unit. Early Hum. Dev..

[17] Kennedy LA (1984). Changes in the term mouse placenta associated with maternal alcohol consumption and fetal growth deficits. Am. J. Obstet. Gynecol..

[18] La Torre R, Nigro G, Mazzocco M, Best AM, Adler SP (2006). Placental enlargement in women with primary maternal cytomegalovirus infection is associated with fetal and neonatal disease. Clin. Infect. Dis..

[19] Mairesse J (2007). Maternal stress alters endocrine function of the feto-placental unit in rats. Am. J. Physiol. Metab..

[20] Pastrakuljic A, Derewlany LO, Koren G (1999). Maternal cocaine use and cigarette smoking in pregnancy in relation to amino acid transport and fetal growth. Placenta.

[21] Zdravkovic T, Genbacev O, McMaster MT, Fisher SJ (2005). The adverse effects of maternal smoking on the human placenta: a review. Placenta.

[22] Mueller BR, Bale TL (2008). Sex-specific programming of offspring emotionality after stress early in pregnancy. J. Neurosci..

[23] Mueller BR, Bale TL (2007). Early prenatal stress impact on coping strategies and learning performance is sex dependent. Physiol. Behav..

[24] Howerton CL, Morgan CP, Fischer DB, Bale TL (2013). O-GlcNAc transferase (OGT) as a placental biomarker of maternal stress and reprogramming of CNS gene transcription in development. Proc. Natl Acad. Sci. USA.

[25] Bond MR, Hanover JA (2013). O-GlcNAc cycling: a link between metabolism and chronic disease. Annu. Rev. Nutr..

[26] Howerton CL, Bale TL (2014). Targeted placental deletion of OGT recapitulates the prenatal stress phenotype including hypothalamic mitochondrial dysfunction. Proc. Natl Acad. Sci. USA.

[27] Chu CS (2014). O-GlcNAcylation regulates EZH2 protein stability and function. Proc. Natl Acad. Sci. USA.

[28] Sanz E (2009). Cell-type-specific isolation of ribosome-associated mRNA from complex tissues. Proc. Natl Acad. Sci. USA.

[29] Sood R, Zehnder JL, Druzin ML, Brown PO (2006). Gene expression patterns in human placenta. Proc. Natl Acad. Sci. USA.

[30] Mao J (2010). Contrasting effects of different maternal diets on sexually dimorphic gene expression in the murine placenta. Proc. Natl Acad. Sci. USA.

[31] Gallou-Kabani C (2010). Sex-and diet-specific changes of imprinted gene expression and DNA methylation in mouse placenta under a high-fat diet. PLoS ONE.

[32] Gabory A (2012). Maternal diets trigger sex-specific divergent trajectories of gene expression and epigenetic systems in mouse placenta. PLoS ONE.

[33] Goldstein JM (2007). Hypothalamic abnormalities in schizophrenia: sex effects and genetic vulnerability. Biol. Psychiatry.

[34] Xu LM (2012). AutismKB: an evidence-based knowledgebase of autism genetics. Nucleic Acids Res..

[35] Mueller BR, Bale TL (2006). Impact of prenatal stress on long term body weight is dependent on timing and maternal sensitivity. Physiol. Behav..

[36] Pankevich DE, Mueller BR, Brockel B, Bale TL (2009). Prenatal stress programming of offspring feeding behavior and energy balance begins early in pregnancy. Physiol. Behav..

[37] Spratt EG (2012). Enhanced cortisol response to stress in children in autism. J. Autism Dev. Disord..

[38] Kurth F (2011). Diminished gray matter within the hypothalamus in autism disorder: a potential link to hormonal effects?. Biol. Psychiatry.

[39] Randazzo WT, Dockray S, Susman EJ (2008). The stress response in adolescents with inattentive type ADHD symptoms. Child Psychiatry Hum. Dev..

[40] Ma L, Chen YH, Chen H, Liu YY, Wang YX (2011). The function of hypothalamus–pituitary–adrenal axis in children with ADHD. Brain Res..

[41] Koolschijn PCMP, van Haren NEM, Pol HEH, Kahn RS (2008). Hypothalamus volume in twin pairs discordant for schizophrenia. Eur. Neuropsychopharmacol..

[42] Comer FI, Hart GW (2001). Reciprocity between O-GlcNAc and O-phosphate on the carboxyl terminal domain of RNA polymerase II. Biochemistry.

[43] Fujiki R (2011). GlcNAcylation of histone H2B facilitates its monoubiquitination. Nature.

[44] Sakabe K, Wang Z, Hart GW (2010). β-N-acetylglucosamine (O-GlcNAc) is part of the histone code. Proc. Natl Acad. Sci. USA.

[45] Zhang S, Roche K, Nasheuer HP, Lowndes NF (2011). Modification of histones by sugar beta-N-acetylglucosamine (GlcNAc) occurs on multiple residues, including histone H3 serine 10, and is cell cycle-regulated. J. Biol. Chem..

[46] Dehennaut V, Leprince D, Lefebvre T (2014). O-GlcNAcylation, an Epigenetic Mark. Focus on the histone code, TET family proteins, and polycomb group proteins. Front. Endocrinol..

[47] Chen Q, Chen Y, Bian C, Fujiki R, Yu X (2013). TET2 promotes histone O-GlcNAcylation during gene transcription. Nature.

[48] Pastor WA, Aravind L, Rao A (2013). TETonic shift: biological roles of TET proteins in DNA demethylation and transcription. Nat. Rev. Mol. Cell Biol..

[49] De Bruyne E (2008). Epigenetic silencing of the tetraspanin CD9 during disease progression in multiple myeloma cells and correlation with survival. Clin. Cancer Res..

[50] Deplus R (2013). TET2 and TET3 regulate GlcNAcylation and H3K4 methylation through OGT and SET1/COMPASS. EMBO J..

[51] Splinter E (2011). The inactive X chromosome adopts a unique three-dimensional conformation that is dependent on Xist RNA. Genes Dev..

[52] Calabrese JM (2012). Site-specific silencing of regulatory elements as a mechanism of X inactivation. Cell.

[53] Dubois A (2014). Spontaneous reactivation of clusters of X‐linked genes is associated with the plasticity of X‐inactivation in mouse trophoblast stem cells. Stem Cells.

[54] Mugford JW (2014). Evidence for local regulatory control of escape from imprinted X chromosome inactivation. Genetics.

[55] Plath K (2003). Role of histone H3 lysine 27 methylation in X inactivation. Science.

[56] Chow J, Heard E (2009). X inactivation and the complexities of silencing a sex chromosome. Curr. Opin. Cell Biol..

[57] Wenzel PL, Leone G (2007). Expression of Cre recombinase in early diploid trophoblast cells of the mouse placenta. Genesis.

[58] Langmead B, Salzberg SL (2012). Fast gapped-read alignment with Bowtie 2. Nat. Methods.

[59] Heinz S (2010). Simple combinations of lineage-determining transcription factors prime cis-regulatory elements required for macrophage and B cell identities. Mol. Cell.

[60] Love MI, Huber W, Anders S (2014). Moderated estimation of fold change and ispersion for RNA-seq data with DESeq2. Genome Biol..

[61] Liao Y, Smyth GK, Shi W (2014). featureCounts: an efficient general purpose program for assigning sequence reads to genomic features. Bioinformatics.

[62] Liao Y, Smyth GK, Shi W (2013). The Subread aligner: fast, accurate and scalable read mapping by seed-and-vote. Nucleic Acids Res..

[63] R Development Core Team R (2011). R: a language and environment for statistical computing. R. Found. Stat. Comput..

[64] Wickham H (2011). The split-apply-combine strategy for data analysis. J. Stat. Softw..

[65] Warnes, G. R. et al. gplots: Various R programming tools for plotting data. R Package version 2 (2009).

[66] Neuwirth, E. RColorBrewer: ColorBrewer palettes. R Package version 1 (2011).

[67] Wilkinson, L. & Urbanek, S. Venneuler: Venn and Euler Diagrams. R package version 1.1-0. Available at http://CRAN.R-project.org/package=venneuler (2011).

[68] Dennis G (2003). DAVID: database for annotation, visualization, and integrated discovery. Genome Biol..

