# Supplementary material for: Placental H3K27me3 establishes female resilience to prenatal insults
Source: Nat Commun. 2018 Jul 2;9:2555. doi: 10.1038/s41467-018-04992-1 (PMC6028627; doi:10.1038/s41467-018-04992-1)
Supplement: Supplementary file 1 — Supplementary Information [file 41467_2018_4992_MOESM1_ESM.pdf]

Supplementary Information

Placental H3K27me3 establishes female resilience to prenatal insults

Nugent *et al.*

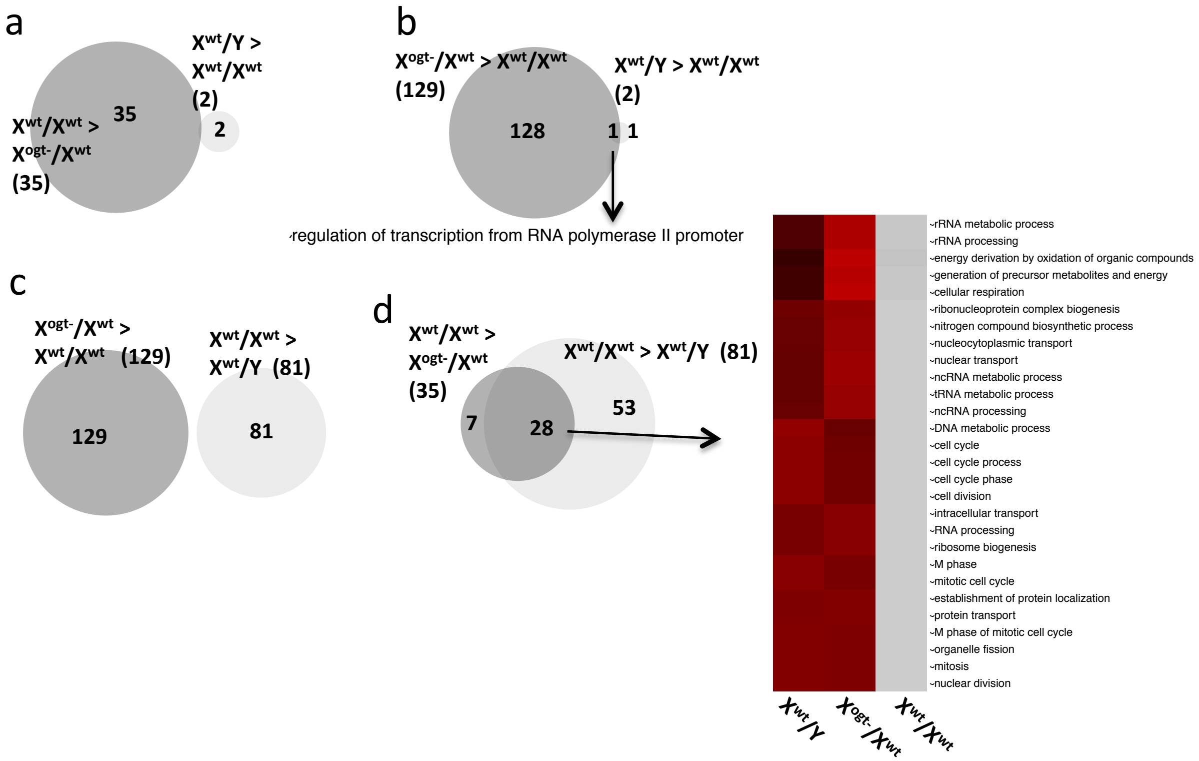

Supplementary Figure 1. Coordinated biological processes associated with differential trophoblast gene expression at E12.5 a, There were no common GO gene sets that were enriched in X^{wt}/X^{wt} relative to X^{ogt-}/X^{wt} and enriched in X^{wt}/Y relative to X^{wt}/X^{wt}. **b**, The GO biological process "regulation of transcription from RNA pol. II promoter" was enriched in both X^{ogt-}/X^{wt} and X^{wt}/Y relative to X^{wt}/X^{wt} trophoblasts. **c**, There were no common GO gene sets that were enriched in X^{ogt-}/X^{wt} relative to X^{wt}/X^{wt} and in X^{wt}/X^{wt} relative to X^{wt}/Y trophoblasts. **d**, 28 GO biological process (displayed in the heatmap) were enriched in X^{wt}/X^{wt} trophoblasts relative to both X^{ogt-}/X^{wt} and X^{wt}/Y. N=3 X^{wt}/Y, n=4 X^{wt}/X^{wt}, and n=5 X^{ogt-}/X^{wt} placentas, from 5 individual litters with n=1/litter/group to control for litter effects.

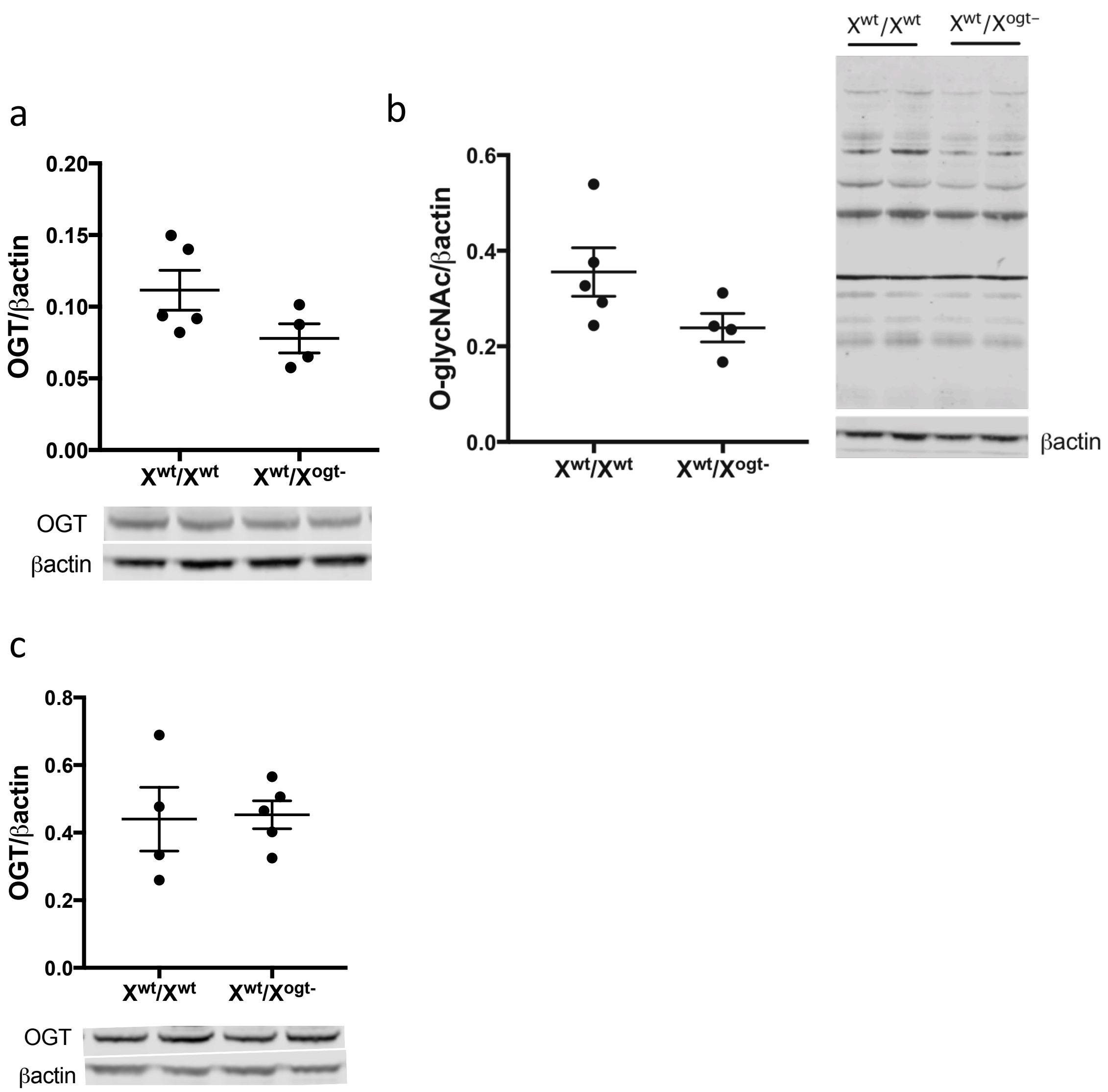

Supplementary Figure 2. Placental-specific OGT reduction. a, Western blot of OGT ($t(7)=1.86$, $p=0.0526$) and **b,** O-glycNAcylated proteins (t-test, $t(7)=1.844$, $p=0.0539$) on whole cell placental lysates in E12.5 trophoblast X^{wt}/X^{wt} and X^{ogt-}/X^{wt} females ($n=5$ X^{wt}/X^{wt}, $n=4$ X^{ogt-}/X^{wt} from 4 litters (a maximum of 2/litter/group to control for litter effects). **c,** OGT in brain whole cell lysates from trophoblast X^{wt}/X^{wt} and X^{ogt-}/X^{wt} females ($t(7)=0.1341$, $p=0.4486$; $n=4$ X^{wt}/X^{wt}, $n=5$ X^{ogt-}/X^{wt} from 4 litters with a maximum of 2/litter/group to control for litter effects). Bars represent mean ± sem.

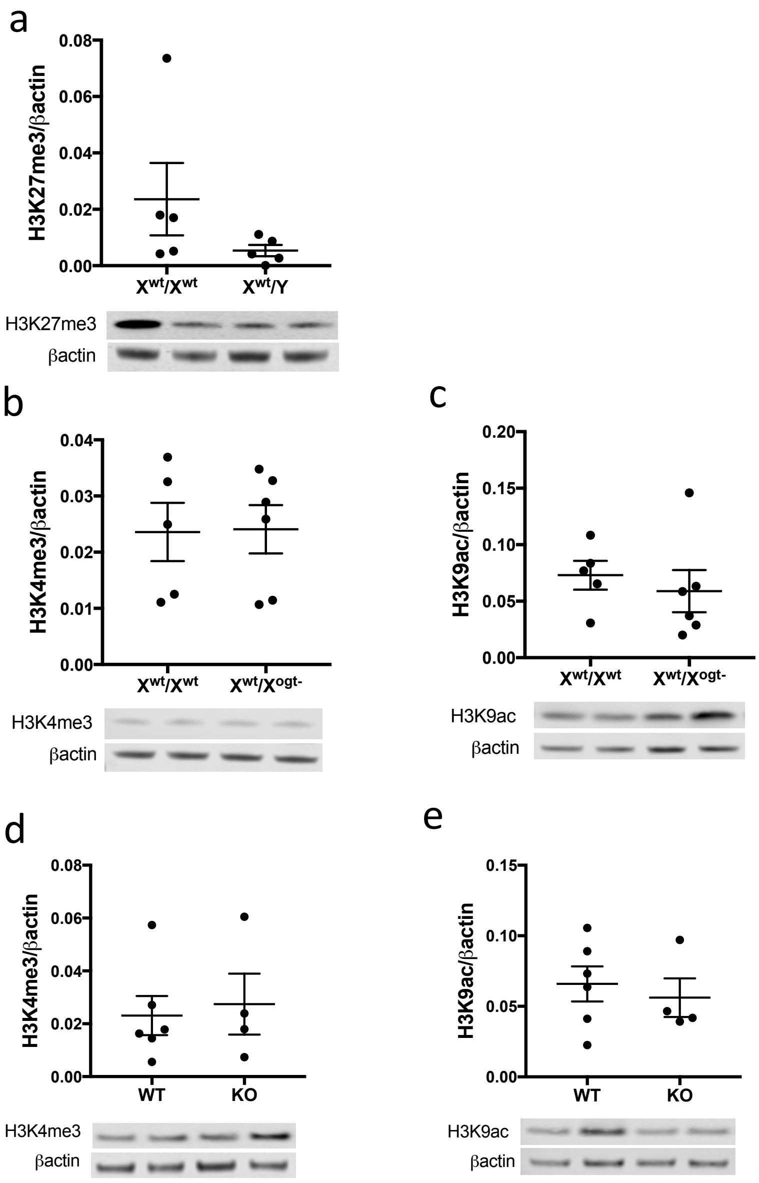

Supplementary Figure 3. Histone quantification in placenta. a, Western blot of H3K27me3 in placental nuclear extracts from E18.5 trophoblast X^{wt}/X^{wt} and X^{ogt-}/X^{wt} females (t-test, t(8)=1.407, p=0.0986; n=5/group from 4 litters with a maximum of 2/litter/group to control for litter effects). **b,** Western blot of H3K4me3 (t(9)=0.07174, p=0.9444) and **c,** H3K9Ac (t(9)=0.5947, p=0.5667) in placental nuclear extracts from E12.5 trophoblast X^{wt}/X^{wt} and X^{ogt-}/X^{wt} females (n=5 X^{wt}/X^{wt}, n=6 X^{ogt-}/X^{wt} from 7 litters (a maximum of 2/litter/group to control for litter effects). **d,** Western blot of H3K4me3 (t(8)=0.3338, p=0.7471) and **e,** H3K9ac (t(8)=0.5148, p=0.6206) in placental nuclear extracts from E12.5 trophoblast WT and *Ezh2* KO females (n=6 WT, n=4 KO from 6 litters with a maximum of 2/litter/group to control for litter effects). Bars represent mean ± sem.

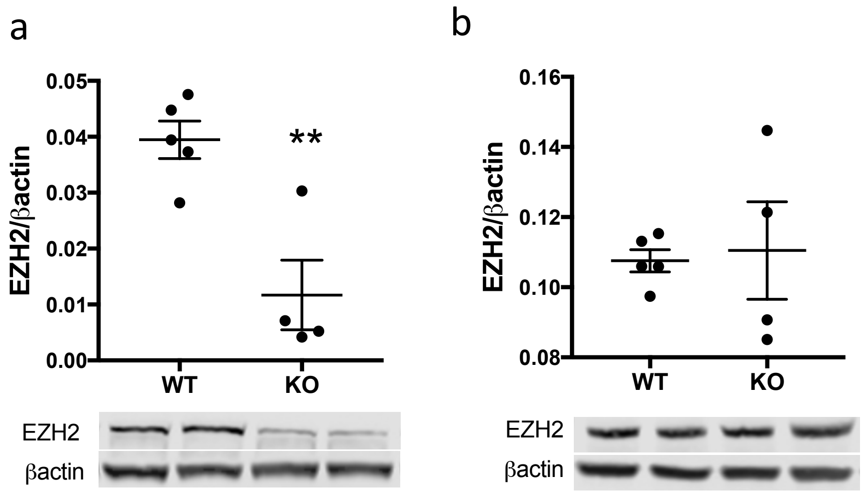

Supplementary Figure 4. Placenta specific EZH2 reduction. a, Western blot of EZH2 in whole cell placenta (t-test, t(7)=4.161, p=0.0021) and **b,** brain (t(7)=0.2296, p=0.4125) lysates from trophoblast WT and *Ezh2* KO E12.5 females (n=5 WT, n=4 KO from 6 litters with a maximum of 2/litter/group to control for litter effects). Bars represent mean ± sem.

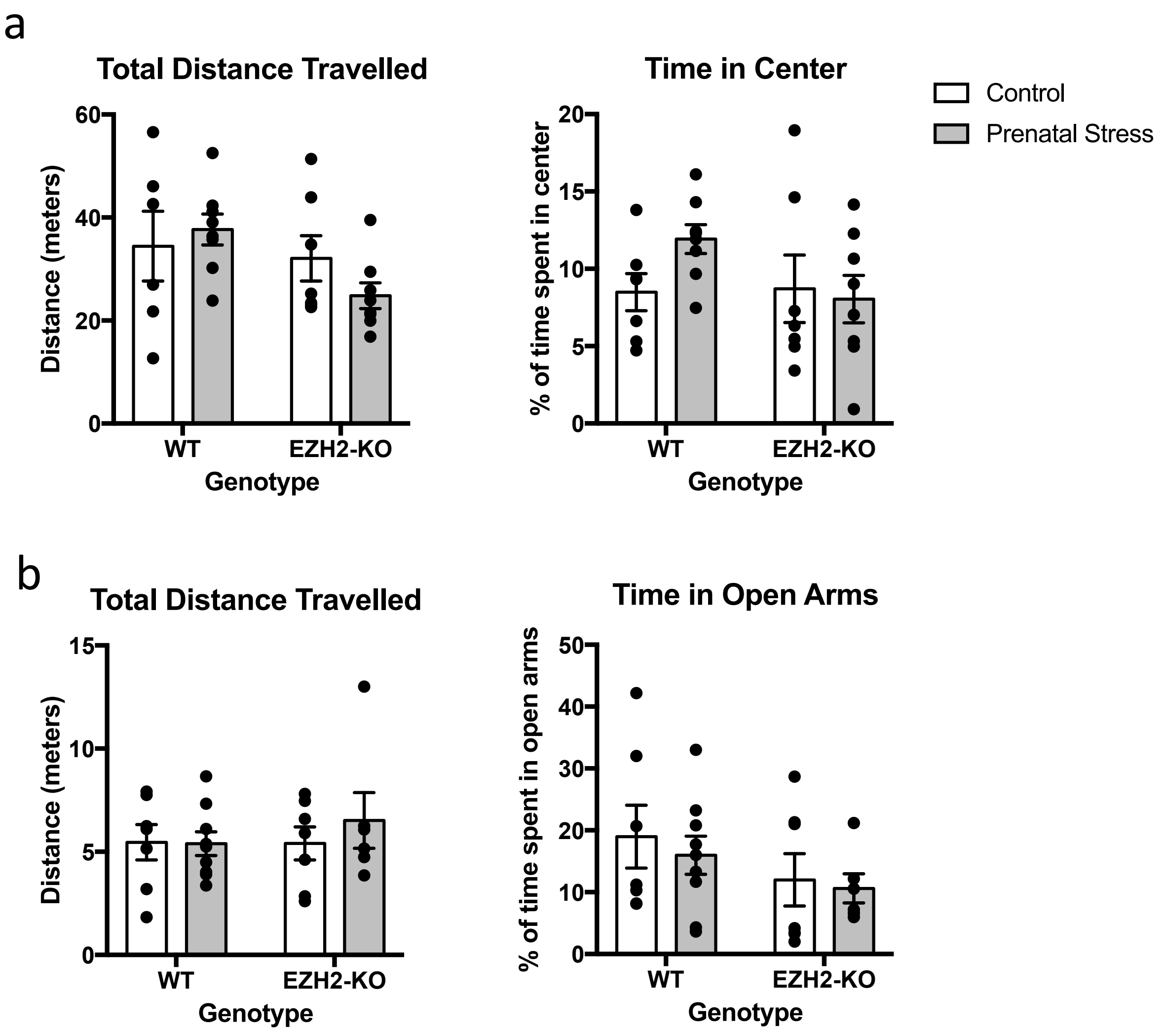

Supplementary Figure 5. Behavioral validation of trophoblast *Ezh2* knockout female mice. a, We found no significant differences in distance travelled (Two-way ANOVA; $F_{treatment(1,25)=}$0.238, p=0.63; $F_{genotype(1,25)=}$3.403, p=0.07, $F_{int(1,25)=}$1.605, p=0.2169) or percentage of time in the center of the open field arena ($F_{treatment(1,25)=}$2.667, p=0.115; $F_{genotype(1,25)=}$0.159, p=0.693, $F_{int(1,25)=}$4.23, p=0.052) **b**, Similarly, we found no differences in distance travelled ($F_{treatment(1,25)=}$0.179, p=0.675; $F_{genotype(1,25)=}$2.008, p=0.1688, $F_{int(1,25)=}$0.006, p=0.936) or percentage of time in open arms in the elevated plus maze. N=7 Con WT, n=9 PS WT, n=7 Con *Ezh2* KO, n=6 PS *Ezh2* KO from 7 PS and 8 Con litters with a maximum of 2/genotype/litter. Bars represent mean ± sem.

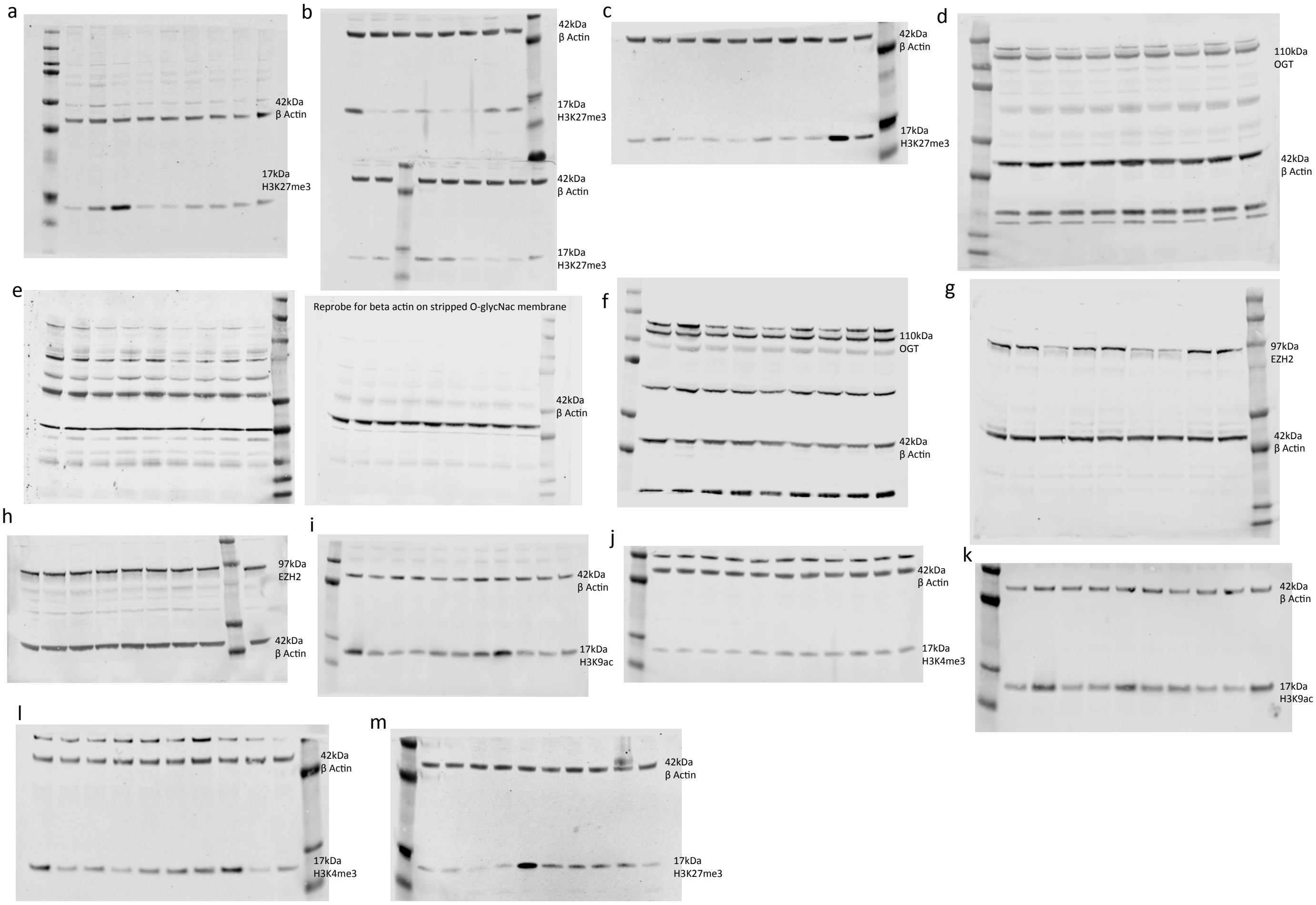

Supplementary Figure 6. Full Western immunoblot images. a, H3K27me3 in term human placenta (fetal side). **b,** H3K27me3 in E12.5 mouse placenta from trophoblast X^{wt}/Y, X^{wt}/X^{wt} and X^{ogt-}/X^{wt}. **c,** H3K27me3 in mouse placenta from E12.5 trophoblast WT and *Ezh2* KO females. **d,** OGT in placentas of E12.5 trophoblast X^{wt}/X^{wt} and X^{ogt-}/X^{wt} females. **e,** Left: O-glycNAc in placentas of E12.5 trophoblast X^{wt}/X^{wt} and X^{ogt-}/X^{wt} females. Right: Beta actin reprobe of membrane used for O-glycNAc quantification. **f,** OGT in brains of trophoblast X^{wt}/X^{wt} and X^{ogt-}/X^{wt} females. **g,** EZH2 in placentas of trophoblast WT and *Ezh2* KO E12.5 females. **h,** EZH2 in brains of trophoblast WT and *Ezh2* KO females. **i,** H3K9ac in placentas of E12.5 trophoblast X^{wt}/X^{wt} and X^{ogt-}/X^{wt} females. **j,** H3K4me3 in placentas of E12.5 trophoblast X^{wt}/X^{wt} and X^{ogt-}/X^{wt} females. **k,** H3K9ac in placentas of E12.5 trophoblast WT and *Ezh2* KO females. **l,** H3K4m3 in placentas of trophoblast WT and *Ezh2* KO E12.5 females. **m,** H3K27me3 in E18.5 X^{wt}/Y and X^{wt}/X^{wt} mouse placenta.
