# Supplementary material for: Placental H3K27me3 establishes female resilience to prenatal insults
Source: Nat Commun. 2018 Jul 2;9:2555. doi: 10.1038/s41467-018-04992-1 (PMC6028627; doi:10.1038/s41467-018-04992-1)
Supplement: Supplementary file 2 — Description of Additional Supplementary Files [file 41467_2018_4992_MOESM2_ESM.pdf]

Description of Additional Supplementary Files

File Name: Supplementary Data 1
Description: Gene ontology (GO) biological processes enriched in gene sets with sex and Ogt-mediated expression differences from trophoblast-specific RNAseq.

File Name: Supplementary Data 2
Description: List of genes with significant sex differences in expression which were significantly altered by placental Ogt reduction in females from trophoblastspecific RNA-seq.

File Name: Supplementary Data 3
Description: List of genes with significant sex differences in expression which were significantly altered by placental Ogt reduction in females from hypothalamic RNA-seq.

File Name: Supplementary Data 4
Description: List of genes significantly altered by exposure to prenatal stress in females with placental Ezh2 reduction from hypothalamic RNA-seq.

File Name: Supplementary Data 5
Description: List of hypothalamic genes with coordinated up or down regulation in wt females vs. males/trophoblast Ogt hemizygous and Ezh2 ko control females vs. Ezh2 ko PS females.

File Name: Supplementary Data 6
Description: List of genes, including chromosome location, altered by trophoblast-specific Ogt reduction from trophoblast-specific RNA-seq.
